# Supplementary material for: A pragmatic reevaluation of the efficacy of nonhuman primate optogenetics for psychiatry
Source: Oxf Open Neurosci. 2022 Apr 29;1:kvac006. doi: 10.1093/oons/kvac006 (PMC10939311; doi:10.1093/oons/kvac006)
Supplement: suppl_data_kvac006 [file suppl_data_kvac006.zip › kvac006_REVIEWER COMMENTS oxfnsc-2021-003.docx]

REVIEWER COMMENTS

OXFNSC-2021-003 A pragmatic reevaluation of the efficacy of nonhuman primate optogenetics for psychiatry

Original submission

Reviewer 1

This paper discusses issues relevant to the application of optogenetics tools, while also commenting on general issues in cross-species translation in neuroscience, in non-human primates. This paper is primarily a response to a report in Neuron by Tremblay et al. (2020), which the authors argue is flawed and may overstate the success of optogenetic tools in non-human primates (reported as 91% in the original report). Given we are almost two decades beyond the initial demonstration of optogenetic manipulation of neural circuits in mice and rats, the current manuscript is of importance in addressing the unspoken question of why optogenetics has not been readily taken up and successful in non-human primates given the ease of its use in rodents - indeed these tools are so readily used they are now considered standard in the rodent neuroscience toolkit. The authors relate this issue to the inevitable concern of the ultimate translational utility of these methods in humans to treat psychiatric illness or neurological disease. While I have concerns that this report can be viewed as a direct reply to the initial paper by Tremblay et al. (2020), the manuscript more generally comments on the utility of neuroscientific advances if they are focused on the incorporation of tools as opposed to fundamental questions about brain and behavior that should hold regardless of species. I found this manuscript timely and provides substantive arguments more generally about the future of behavioral neuroscience. I mainly have concerns that the authors may misrepresent the utility of optogenetic investigations in rodents and hope that these serve to refine the authors’ arguments. Overall, this a refreshing perspective on the state of neuroscience on which I have only a few comments for the authors to consider.

1) The authors should more seriously consider whether the translational goal of optogenetics is to directly adapt the method to be used as in model species to be used in humans (e.g. as stated in line 90) or to inform or modify treatment approaches available in humans to therapeutic benefit (e.g. using optogenetic investigation to inform modified approaches to DBS as in Creed, Pascoli, Luscher 2015 Science). The introduction seems to imply that rodent neuroscientists see the ultimate vision of their work as humans with fiber optic cannulas and LEDs implanted, which I think is a mischaracterization. Certainly many rodent optogenetic studies are overly reductive, their claims overstated, and the continued use of poor behavioral approaches will limit the lasting impact of these findings – yet this could be the topic of separate a review. I am not of the impression that in their current form any viral-based tool will ultimately be successful in humans for various reasons or that neuroscientists are naïve in this sense, but that does not prevent insights gained from these tools in model species as being beneficial. There is some reference to these sorts of ideas in the final sentence of the primary results but the introduction is relatively damning in tone for rodent neuroscience.
2) While enjoyable to read, the frequent incorporation of terminology from Figure 1 about hype cycles made it difficult at times to parse the argument being made – especially towards the conclusion of the manuscript. Though not necessary, I think lessening the heavy-handed use of hype cycle comments and terminology would increase the readability of the manuscript.
3) I found it hard to visualize and keep track of the various overlaps of successful experiments for the given categories and conjunctions (behavioral effect given physiology effect, etc.) in the results that form the crux of the authors’ argument. The circle plots of the three categories are excellent in Fig. 2 but these are not, at least in my reading, the main argument of the current paper (I agree that histological verification alone is not a demonstration of success of optogenetics or any other viral based tool) and an additional visualization (stacked bar proportions, Venn diagram, etc.) would be of great assistance for the conjunctions. Even adding to Fig. 2 and any potential subsequently produced figures the findings of success by Tremblay’s definition would be useful or some diagram depicting how Tremblay defined success/experiments and the current approach for defining an experiment. For instance, I missed what the percent success would be if injections (Tremblay) and not experiment (current paper) was used for behavior given histology or behavior given neurophysiology for both weak and strong success.
4) On Line 101, “First, lissencephalic mouse brains are structured such that many of the important neural hubs for behavior, generally, and for neuropsychiatric disease, specifically, are on the surface of the brain which means that they are easier to access both with the virus to transfect neurons and the light needed to modify neural activity.” This is a bold statement that implies the mouse/rat dorsal cortical sheet containing mainly sensory cortical areas is the most important for neuroscientific investigations which would require citations to support this claim. Do the authors instead mean to refer to the smaller size of rodent brains ~6mm deep for mice and ~11mm for rats making it easier to access virtually any brain structure of interest? This is particularly confusing given the later statements regarding the successes and failures of non-human primate optogenetics in primarily sensory cortical areas that are near the surface of the non-human primate brain as well.
5) On Lines 49 and 96 the authors state “Originally developed in mice,” in regard to optogenetic tools. This is not necessarily accurate. These are modified algal, bacterial, archaeal, etc. proteins that were first demonstrated to be successful in cultured rat hippocampal neurons (at least for Channelrhodopsin-2; or even earlier reports by Miesenböck in Drosophila for the actual use of such an approach to modify neurons and behavior with light) and then ultimately in awake-behaving mice with extensive work to make this tool appropriate for use in mice and rats. The authors should be more explicit about the ease of application of optogenetics in rodent behavioral neuroscience or reframe this sentence to more appropriately reflect the development of optogenetics.
6) Baxter and Costa 2021 is missing from the references.

Reviewer 2

I read this paper briefly as I feel that the authors should have a right to respond to our papers as they wish (in Neuron), and I am aware that in Neuron their response were ignored, so I think these authors should voice their views somewhere, and why not in this fine Journal! 

So in all, I read this paper pretty briefly and will give the Authors my suggestions which they can choose to ignore. 

My suggestions (in terms of science)

The fact is that optogenetics works well in many brain areas if done correctly in NHPs (see recent papers from the Hikosaka lab that have used it with great success to interrogate circuits and behavior). The key point is who does it and how – and yes that means the method is subtle and yes that is how most biological methods are (somehow, single unit ephys – at least in cortex - is robust as are lesion work; I mean robust to any operator or user, which does not mean they are better or more insightful, it just means they are less artisanal, or difficult). Many factors depend on success as the authors know themselves, and I think they need to spend more time carefully separating different papers from each other and each work's unique contributions and failure. I have the same comment for the Neuron paper they are commenting on. Virus types, injection speeds, how the virus is handled, how it is injected (see individual comments in original Neuron Paper from individual labs) all matter (they matter very much even in rodents, where the artisanal nature of techniques is assumed). So, on average, I would implore the authors to be cautious, conservative, and pay attention to details of individual papers. But ultimately, I am not going to enforce any of this on this publication. And in Costa and Baxter’s defense, a grand-average-analysis of opto (as was done in Neuron paper) is a bit odd if not useful from my perspective. 
But, gosh, if we did that for antidromic stimulation, a method that has given us a huge amount of knowledge, we would conclude that it is utterly useless! So, again, be careful!

Cavanaugh and Monosov, et al. (btw, not from 2021) – the first to directly manipulate motor systems with opto - carefully estimated the amount of tissue one needs to mediate with opto and how opto works (and the message was cautioning for sure, including for mechanism itself, not just with regard to the tissue volume and expression levels). The message was NOT that the SC is the only area that can be modulated, but rather that for certain questions opto is useful and for others not. And this depends on brain area (cell types, organization, size), and question at hand! E.g., recent Hikosaka papers show opto works great. But those are golden hands and choosing the right question in the right brain area, and using the right virus for the question (which btw, many NHP groups have not carefully worried about). But being careful is required for ANY sophisticated method not just opto. Is failure rates that interesting with that perspective in mind?

My suggestions (in terms of positioning)

I would not use inflammatory terms like “hype cycle” and stick to the data. I agree many opto papers across many species are cheap, use cheap or poor behavioral control, and provide little insight. But this is NOT opto specific. So, I would encourage civility. Hype is in the eye of the beholder. 

I would not pitch the entire thing in terms of “translation”. The Optogenetic Neuron paper was more about using optogenetics to study circuits in NHPs than anything else. The translational stuff to humans is not exactly a key point. And comparing brain sizes, as the authors do is misleading. Some brain areas are big, some are small, some expanded during evolution, some did not. I am not sure if this is a key issue for opto, because for example some brain areas remain small in NHP. Also, opto arrays can be made to illuminate lots of tissue in primates. I think the challenges of opto are so much richer. If it was me, I would leave the human translation alone. For example, are the authors also going to talk about the immunogenicity and other potential toxic affects of AAVs in detail and more broadly? (e.g. some people have recently found AAVs can cause degeneration of D.gyrus, but the mechanism is unclear, that data is making way through reviews, i believe). If the authors want, they can attack more effectively on cell type differences and organization within brain area. But, in any case, I would shift a bit from translation. 

In general, the authors ought not to worry about my views here and can go ahead with their paper. I will not censor them. I just think a more subtle gentle handling is in order.

Decision letter

20-Jan-2022 

Dear Reviewer, 

Thank you for your recent assistance in evaluating this manuscript.  The editor made the following decision Minor Revision.  The reviewer comments are available at the end of this email.   

Thank you for your kind support of our peer review process. 


Sincerely, 
Dr. Alicia Izquierdo 
Editor, Oxford Open Neuroscience 
aizquie@psych.ucla.edu 

Associate Editor

Author response

Please see our responses to the editor’s feedback and the concerns/points made by the reviewers, indented, after the specific comments from the editor and reviewers, below. 

Both reviewers found this work timely and important. There was also substantial overlap and agreement in their suggestions, which I summarize here, as three main points. Your manuscript should become acceptable pending satisfactory responses to these three essential revisions: 
1) Both reviewers agreed that the use of ‘hype cycles’ was distracting and/or inflammatory. Authors are encouraged to use a different approach to communicate the cycle of interest and successful deployment of new technologies in nonhuman primate vs. rodent brains, more generally. 

Although we think that this concept might correctly describe some of the behavior in the field, we appreciate and agree that this framing is potentially inflammatory and have removed it from the revised manuscript. The main points of the paper can be made without reference to the concept of a "hype cycle" and we have eliminated it from the new draft.  

2) Both reviewers also recommended to more seriously consider (re-evaluate) whether the goal of optogenetics is to directly adapt the method in model systems to human brains. 

We appreciate this point and have tried to draw finer distinctions in the revised manuscript. Nevertheless, we do not wish to minimize the significant challenges that remain for using optogenetic (and other novel neuromodulation technologies) in the domain of psychiatric neuroscience. This is not the only goal of these approaches, but it clearly articulated as a primary goal in Tremblay et al. (2020). 

3) Reviewer 1 suggested to clarify the relevance of superficial cell layers in optogenetic studies. Reviewer 2 had a similar concern about comparing brain sizes. Taken together, the authors should clarify the ‘ease of access’ argument across rodent and primate species. 

We regret the confusion about these points. Following the issues raised by Reviewer 1, our first discussion of this issue was meant in part to refer to absolute differences in brain size, whereas the second point in the context of sensory/motor areas was related to the complexity of affecting behavior as well as with the surgical approach. We have modified the text accordingly to clarify these points; the position of a target region on the surface of a brain is by no means a guarantee of success in a larger primate brain. 

Please address these three main points above, in addition to Reviewer 1’s points 5 and 6 below, and when you resubmit, briefly describe your revisions in the Author Response to Reviewers file and also indicate the changes in the text. I include the Reviewers’ comments below, which offer additional thoughts and suggestions for improvement which you can incorporate in your revision as you see fit. 

We have removed the statement about optogenetics being "developed in mice" (point 5) and included the Baxter and Costa (2021) reference (point 6) in the revised manuscript.

Once you resubmit, this manuscript will undergo rapid editorial review, and I will inform you promptly of a decision. Of course, you may also contact me directly if there are reasons why more time is needed to complete the revision, and we can extend the deadline. 

REVIEWER 1 
This paper discusses issues relevant to the application of optogenetics tools, while also commenting on general issues in cross-species translation in neuroscience, in non-human primates. This paper is primarily a response to a report in Neuron by Tremblay et al. (2020), which the authors argue is flawed and may overstate the success of optogenetic tools in non-human primates (reported as 91% in the original report). Given we are almost two decades beyond the initial demonstration of optogenetic manipulation of neural circuits in mice and rats, the current manuscript is of importance in addressing the unspoken question of why optogenetics has not been readily taken up and successful in non-human primates given the ease of its use in rodents - indeed these tools are so readily used they are now considered standard in the rodent neuroscience toolkit. The authors relate this issue to the inevitable concern of the ultimate translational utility of these methods in humans to treat psychiatric illness or neurological disease. While I have concerns that this report can be viewed as a direct reply to the initial paper by Tremblay et al. (2020), the manuscript more generally comments on the utility of neuroscientific advances if they are focused on the incorporation of tools as opposed to fundamental questions about brain and behavior that should hold regardless of species. I found this manuscript timely and provides substantive arguments more generally about the future of behavioral neuroscience. I mainly have concerns that the authors may misrepresent the utility of optogenetic investigations in rodents and hope that these serve to refine the authors’ arguments. Overall, this a refreshing perspective on the state of neuroscience on which I have only a few comments for the authors to consider. 

1) The authors should more seriously consider whether the translational goal of optogenetics is to directly adapt the method to be used as in model species to be used in humans (e.g. as stated in line 90) or to inform or modify treatment approaches available in humans to therapeutic benefit (e.g. using optogenetic investigation to inform modified approaches to DBS as in Creed, Pascoli, Luscher 2015 Science). The introduction seems to imply that rodent neuroscientists see the ultimate vision of their work as humans with fiber optic cannulas and LEDs implanted, which I think is a mischaracterization. Certainly many rodent optogenetic studies are overly reductive, their claims overstated, and the continued use of poor behavioral approaches will limit the lasting impact of these findings – yet this could be the topic of separate a review. I am not of the impression that in their current form any viral-based tool will ultimately be successful in humans for various reasons or that neuroscientists are naïve in this sense, but that does not prevent insights gained from these tools in model species as being beneficial. There is some reference to these sorts of ideas in the final sentence of the primary results but the introduction is relatively damning in tone for rodent neuroscience. 
2) While enjoyable to read, the frequent incorporation of terminology from Figure 1 about hype cycles made it difficult at times to parse the argument being made – especially towards the conclusion of the manuscript. Though not necessary, I think lessening the heavy-handed use of hype cycle comments and terminology would increase the readability of the manuscript. 

We have addressed these points in editorial recommendation 1, above. 

3) I found it hard to visualize and keep track of the various overlaps of successful experiments for the given categories and conjunctions (behavioral effect given physiology effect, etc.) in the results that form the crux of the authors’ argument. The circle plots of the three categories are excellent in Fig. 2 but these are not, at least in my reading, the main argument of the current paper (I agree that histological verification alone is not a demonstration of success of optogenetics or any other viral based tool) and an additional visualization (stacked bar proportions, Venn diagram, etc.) would be of great assistance for the conjunctions. Even adding to Fig. 2 and any potential subsequently produced figures the findings of success by Tremblay’s definition would be useful or some diagram depicting how Tremblay defined success/experiments and the current approach for defining an experiment. For instance, I missed what the percent success would be if injections (Tremblay) and not experiment (current paper) was used for behavior given histology or behavior given neurophysiology for both weak and strong success. 

We added a figure to clarify the conjunctions (new Figure 2). With regard to conjunctions with the per-injection definition, this was not a comparison Tremblay et al. (2020) used in their paper, but one we carried out to emphasize the importance of considering "success" in the context of behavior. Calculating per-injection success rates on the data set results in per-injection success rates of less than 12%. 

4) On Line 101, “First, lissencephalic mouse brains are structured such that many of the important neural hubs for behavior, generally, and for neuropsychiatric disease, specifically, are on the surface of the brain which means that they are easier to access both with the virus to transfect neurons and the light needed to modify neural activity.” This is a bold statement that implies the mouse/rat dorsal cortical sheet containing mainly sensory cortical areas is the most important for neuroscientific investigations which would require citations to support this claim. Do the authors instead mean to refer to the smaller size of rodent brains ~6mm deep for mice and ~11mm for rats making it easier to access virtually any brain structure of interest? This is particularly confusing given the later statements regarding the successes and failures of non-human primate optogenetics in primarily sensory cortical areas that are near the surface of the non-human primate brain as well. 

As addressed above (response to editor), we have modified the text associated with this point. 

5) On Lines 49 and 96 the authors state “Originally developed in mice,” in regard to optogenetic tools. This is not necessarily accurate. These are modified algal, bacterial, archaeal, etc. proteins that were first demonstrated to be successful in cultured rat hippocampal neurons (at least for Channelrhodopsin-2; or even earlier reports by Miesenböck in Drosophila for the actual use of such an approach to modify neurons and behavior with light) and then ultimately in awake-behaving mice with extensive work to make this tool appropriate for use in mice and rats. The authors should be more explicit about the ease of application of optogenetics in rodent behavioral neuroscience or reframe this sentence to more appropriately reflect the development of optogenetics. 

We have removed the text in question. 

6) Baxter and Costa 2021 is missing from the references. 

This has been added to the reference section. 

REVIEWER 2 
My suggestions (in terms of science) 

The fact is that optogenetics works well in many brain areas if done correctly in NHPs (see recent papers from the Hikosaka lab that have used it with great success to interrogate circuits and behavior). The key point is who does it and how – and yes that means the method is subtle and yes that is how most biological methods are (somehow, single unit ephys – at least in cortex - is robust as are lesion work; I mean robust to any operator or user, which does not mean they are better or more insightful, it just means they are less artisanal, or difficult). Many factors depend on success as the authors know themselves, and I think they need to spend more time carefully separating different papers from each other and each work's unique contributions and failure. I have the same comment for the Neuron paper they are commenting on. Virus types, injection speeds, how the virus is handled, how it is injected (see individual comments in original Neuron Paper from individual labs) all matter (they matter very much even in rodents, where the artisanal nature of techniques is assumed). So, on average, I would implore the authors to be cautious, conservative, and pay attention to details of individual papers. But ultimately, I am not going to enforce any of this on this publication. And in Costa and Baxter’s defense, a grand-average-analysis of opto (as was done in Neuron paper) is a bit odd if not useful from my perspective. 
But, gosh, if we did that for antidromic stimulation, a method that has given us a huge amount of knowledge, we would conclude that it is utterly useless! So, again, be careful!

Cavanaugh and Monosov, et al. (btw, not from 2021) – the first to directly manipulate motor systems with opto - carefully estimated the amount of tissue one needs to mediate with opto and how opto works (and the message was cautioning for sure, including for mechanism itself, not just with regard to the tissue volume and expression levels). The message was NOT that the SC is the only area that can be modulated, but rather that for certain questions opto is useful and for others not. And this depends on brain area (cell types, organization, size), and question at hand! E.g., recent Hikosaka papers show opto works great. But those are golden hands and choosing the right question in the right brain area, and using the right virus for the question (which btw, many NHP groups have not carefully worried about). But being careful is required for ANY sophisticated method not just opto. Is failure rates that interesting with that perspective in mind? 

We appreciate these considerations and in part share them. We have tried to clarify the distinction between utility in a basic research context and translation - although Tremblay et al. (2020) make some fairly strong statements about translation that, at best, could be seriously misinterpreted and at worst are badly misleading, in our opinion. Also, we have updated the Cavanaugh reference – its publication year is 2012. 

My suggestions (in terms of positioning) 

I would not use inflammatory terms like “hype cycle” and stick to the data. I agree many opto papers across many species are cheap, use cheap or poor behavioral control, and provide little insight. But this is NOT opto specific. So, I would encourage civility. Hype is in the eye of the beholder. 

We have removed discussion of the Hype Cycle. 

I would not pitch the entire thing in terms of “translation”. The Optogenetic Neuron paper was more about using optogenetics to study circuits in NHPs than anything else. The translational stuff to humans is not exactly a key point. And comparing brain sizes, as the authors do is misleading. Some brain areas are big, some are small, some expanded during evolution, some did not. I am not sure if this is a key issue for opto, because for example some brain areas remain small in NHP. Also, opto arrays can be made to illuminate lots of tissue in primates. I think the challenges of opto are so much richer. If it was me, I would leave the human translation alone. For example, are the authors also going to talk about the immunogenicity and other potential toxic affects of AAVs in detail and more broadly? (e.g. some people have recently found AAVs can cause degeneration of D.gyrus, but the mechanism is unclear, that data is making way through reviews, i believe). If the authors want, they can attack more effectively on cell type differences and organization within brain area. But, in any case, I would shift a bit from translation. 

As discussed in the response to the editor, we have clarified this discussion and emphasized the claims made by the Tremblay paper in terms of translation. 

In general, the authors ought not to worry about my views here and can go ahead with their paper. I will not censor them. I just think a more subtle gentle handling is in order

Immediate accept on revision

AE comments: I think you have well-addressed the comments raised in review.

Decision letter

08-Mar-2022 

Dear Dr. Bliss Moreau, 

It is a pleasure to accept your manuscript entitled "A pragmatic reevaluation of the efficacy of nonhuman primate optogenetics for psychiatry" in its current form for publication in the Oxford Open Neuroscience. I think you have well-addressed the comments raised in review. 

Thank you for your exciting contribution! On behalf of the Editors of the Oxford Open Neuroscience, we look forward to your continued contributions to the Journal. 

Sincerely, 
Dr. Alicia Izquierdo 
Senior Editor, Oxford Open Neuroscience 
aizquie@psych.ucla.edu 
